# Association between the nutritional quality of Canadian packaged foods and their prices: an analysis across five food categories

**DOI:** 10.1017/S1368980025100797

**Published:** 2025-08-13

**Authors:** Isabelle Petitclerc, Sonia Pomerleau, Laure Saulais, Geneviève Mercille, Marie-Ève Labonté, Véronique Provencher

**Affiliations:** 1 Centre de Recherche Nutrition, Santé et Société (NUTRISS), Institute of Nutrition and Functional Foods (INAF), Université Laval, 2440, Boulevard Hochelaga, Quebec City, QC G1V 0A6, Canada; 2 School of Nutrition, Université Laval, 2440, Boulevard Hochelaga, Quebec City, QC G1V 0A6, Canada; 3 Department of Nutrition, Université de Montréal, 2450 Chemin de la Côte-Sainte-Catherine, Montréal, QC H3T 1A8, Canada; 4 Centre de recherche en santé publique (CReSP), CIUSSS du Centre-Sud-de-l’Île-de-Montréal et Université de Montréal, 2099 Rue Alexandre-DeSève, Montréal, QC H2L 2W5, Canada; 5 Department of Agri-food Economics and Consumer Science, Université Laval, 2440, Boulevard Hochelaga, Quebec, QC G1V 0A6, Canada

**Keywords:** Nutritional quality, Food prices, Food policies, Front-of-packaging labeling, Health inequalities

## Abstract

**Objective:**

To evaluate the association between nutritional quality and food prices within the same food category by: (1) identifying price differences among products above or below the nutrient thresholds of the Canadian front-of-package nutrition symbol and (2) investigating price differences among products with differing numbers of nutrients exceeding these thresholds.

**Design:**

This study is part of the Food Quality Observatory’s work, Québec (Canada).

**Setting:**

For each product, nutrients exceeding the thresholds for Na, sugars and saturated fat were calculated according to Health Canada’s guidelines. Prices per 100 g and per 100 kcal (418 kJ) were calculated. Statistical analyses were performed using RStudio to evaluate the association between these price metrics and nutritional quality, based on these thresholds.

**Participants:**

Five food categories were analysed: sliced breads (*n* 340), breakfast cereals (*n* 392), salty snacks (*n* 569), cookies (*n* 694) and processed cheeses (*n* 118).

**Results:**

Results indicate that nutrient type mediates the association between price and nutritional quality. Products exceeding the saturated fat threshold were generally more expensive, whereas those with elevated sugars and Na contents were cheaper. Products with two nutrients exceeding thresholds tended to cost less than those with one or no nutrient above thresholds. Notably, these results varied within each food category. These patterns varied across food categories.

**Conclusion:**

Foods high in nutrients of concern are typically cheaper within their category, except those high in saturated fat. Findings highlight the importance of monitoring food prices, especially as Canada’s nutrition symbol policy becomes mandatory, to prevent worsening health inequalities.

Ultra-processed foods (UPF) now dominate the food supply in high-income countries, including Canada^(1)^. These products are typically high in energy, have a high glycaemic load and are low in dietary fiber, micronutrients and phytochemicals, while being rich in unhealthy fats, sugars and Na^(2)^. Consequently, the consumption of UPF is associated with an increased risk of obesity, metabolic syndrome, and all-cause mortality^(3)^. Additionally, UPF are often highly palatable, convenient, shelf-stable, and affordable, making their complete removal from consumption habits unrealistic^(4)^. However, nutritional quality of processed foods can vary considerably, even within the same food category^(5)^. Therefore, while it is crucial to continue interventions aimed at reducing UPF consumption, efforts to encourage the selection of healthier options within UPF could also be beneficial^(6)^. However, selecting healthier options within UPF food categories can be challenging due to various factors influencing food choices, such as price, taste, habits and availability, as well as the limited effectiveness of the traditional Nutritional Facts table, due to time constraints and lack of nutrition knowledge^(7,8)^.

Consequently, front-of-packaging labeling (FOPL) is increasingly recognised as an effective public health strategy. Its logos and colors help convey easy-to-understand nutrition information to consumers leading to healthier food choices for people of all socioeconomic status (SES)^(9)^. In this context, Canadian prepackaged food products are expected to increasingly feature FOPLs, as a new nutrition labeling regulation introduced by Health Canada took effect in July 2022^(10)^. Most prepackaged foods exceeding specific thresholds for saturated fat, Na and/or sugars content will be required by January 1st 2026 to display a nutrition symbol on the front of the package, indicating that the product is high in one or more of these nutrients^(10)^. The objectives of the FOPL policy are not only to guide the consumer in making healthier food choices but also to encourage the industry to reformulate their products to offer healthier options^(11)^. However, reformulation is a time-consuming process that often requires a change of ingredients, recipes and/or processing methods, labor for the reformulation process, production of new food packaging, and more, all of which can represent a significant cost for the industry^(12)^. As a result, the industry’s response to these increased costs may involve raising the price of improved food products. However, a study conducted in Chile following the implementation of a FOPL contradicts this hypothesis, as it found no clear evidence of price changes during the first year and a half of implementation^(13)^. Nonetheless, the impact of reformulation in response to food policies on food prices remains understudied and has to be confirmed.

Food price is a crucial element to consider when planning nutrition interventions, as it significantly influences food choices, especially among lower-income households^(14)^. Notably, food price plays a critical role in consumer decision-making when selecting products within a given food category^(15)^. While FOPL provides useful nutritional information, price is likely to remain the leading factor in food choices, especially in today’s economic climate, where food prices have surged in recent years^(16)^. In this context, consumers may face two scenarios. In cases where FOPL and price align, such as when a product is labeled with a nutritional symbol and is more expensive than the other alternatives, the nutrition symbol may strengthen the incentive to avoid that product. Conversely, when FOPL and price diverge, such as when a product with a nutrition symbol is the cheapest option, consumers risk choosing the less healthy alternative due to its lower cost. Indeed, in an experimental marketplace study, it was observed that nutrition labeling on sugary drinks encouraged selection of drinks with less free sugars, but this was not statistically significant. However, as prices increased, participants were significantly less likely to select a sugary drink, indicating that while FOPL could help make healthier food choices, price remains the dominant influence in their decision-making process^(17)^.

Currently, there is a lack of research on the association between retail food prices and their nutritional quality within specific food categories. Indeed, it is well established that higher diet quality is associated with higher diet costs^(18,19)^. However, these studies remain broad and rarely examine specific food categories or individual products. This gap in literature limits our understanding of how prices vary within the same food category between products of differing nutritional quality. A deeper understanding of the association between price and nutritional quality at the product level is crucial, as it could reveal an important barrier consumers face when having to select healthier options, even when tools such as FOPL are available. Investigating this association could uncover inequalities in access to healthier foods, highlighting the need for pricing interventions such as subsidies on healthier food products and taxes on unhealthy ones. This knowledge could better support FOPL and other nutrition policies in inciting healthier food choices and improving diet quality.

Consequently, one may wonder if, in Canada, the prices of prepackaged food products within the same category are associated with their level of healthiness, as indicated by whether they meet FOPL thresholds. Specifically, we aim (1) to explore the potential price differences between food products exceeding or below nutrient thresholds within the same food category and (2) to investigate price differences between products with a different number of nutrients (zero, one, two, or three) exceeding thresholds. We hypothesise that, within a food category, products exceeding thresholds have a lower cost compared to those below these thresholds. To our knowledge, no studies have previously looked at the price and nutritional value of food products in Canada, enhancing the need to assess this aspect.

## Methods

### Setting

The current study is part of the ongoing work of the Food Quality Observatory^(20)^, created in 2016 to monitor the quality of the food supply in the province of Québec. Fifteen prepackaged food categories were initially selected based on their public health relevance^(21)^. Data collection started in 2016 for selected food categories, and by 2022, all 15 had been analysed^(5)^. Every five years, data collection for each food category is repeated to ensure follow-up. Data collection was done in person by purchasing all items from food categories in supermarkets (e.g. Metro, IGA), wholesale stores (e.g. Walmart, Costco) and specialty grocery stores (e.g. Avril, Rachelle-Béry) from Québec City and its surroundings (Canada)^(21)^. The only exception was for salty snacks, partly collected from retailer websites during the COVID-19 pandemic. All package information (e.g. nutrition facts table, serving size, FOPL, etc.) was double-coded in the database by two independent coders to ensure accuracy. Prices observed across the different stores visited were also recorded.

### Analysed food categories

The five food categories analysed were: ready-to-eat (RTE) breakfast cereals, cookies, processed cheeses, sliced breads and salty snacks. These categories were selected from the Food Quality Observatory’s initial report, as they were identified as the ones contributing the most to Na, sugars, or saturated fat purchases among the Québec population^(5)^. Data collection took place in 2021 for breakfast cereals and sliced breads, in 2019 for cookies and in 2020 for processed cheeses and salty snacks. Products within each food category were then grouped through double-coding into different subcategories of similar types to deepen our analyses. For instance, breakfast cereals were categorised into granola, sweetened cereals, muesli, plain, chocolate and bitesize. Detailed criteria used for establishing subcategories are presented in the methodology of the initial portraits of our sectoral studies^(22–26)^. Definitions of these subcategories are available in online Supplemental materials.

### Price metrics

Prices for each food item were calculated using the mean of prices observed across the different stores visited. Different pricing metrics were used in our analysis. Indeed, the choice of price metric has been shown to impact which food is considered the most expensive^(27)^. Price per unit of mass is preferred when comparing prices of similar foods that serve the same dietary purpose^(27)^. Also, using energy as a price metric is most suitable when comparing prices in a food security context, as it relates to the cost of achieving energy balance^(27)^. Food choices based on price per calorie are considered the most representative of actual consumption patterns among low-socioeconomic groups, making it a reliable metric for this population^(28)^. Given the lack of consensus on which price metrics to use when studying food prices, our analyses employed both. All price metrics were calculated using a simple ratio calculation method.

### Nutritional quality assessment: Canadian nutrition symbol

This study was conducted before the official implementation of the front-of-package nutrition label regulation, which will become mandatory by 2026^(10)^. Thus, the analysis of the nutritional quality of food items was based on calculations using the thresholds established for the Canadian nutrition symbol, rather than the actual presence of the nutrition symbol on packaging. For most prepackaged food products, a nutrition symbol will be mandatory when the amount of saturated fat, sugars and/or Na is equal to or greater than 15 % of the Daily Values (DV) recommended for these nutrients. However, if the reference amount for the product is equal to or less than 30 g or 30 ml, the symbol will be required when the amount of each of these three nutrients equals or exceeds 10 % of the DV threshold. Also, in a main dish product with a reference amount ≥ 200 g, the threshold not to exceed is fixed at 30 % of the DV. Nutritional data from our database combined with regulations concerning the nutrition symbol were used to ensure that the specific precision requirements of each product category were adequately followed when needed^(29)^. We then determined which nutrients would be featured in the nutrition symbol for each product if its composition remained unchanged until 2026.

### Statistical analyses

RStudio 4.2.2 was used to perform all statistical analyses. We conducted Wilcoxon tests to compare the prices of individual products based on whether they were above or below the thresholds for Na, saturated fat, or sugars established for the nutrition symbol policy. We did these analyses for both price per 100 g and per 100 kcal (418 kJ). Non-parametric tests were used due to the non-normal distribution of prices across our price metrics within food categories. Additionally, to examine the association between the price of a food product and the number of nutrients exceeding the thresholds, we performed a Kruskal-Wallis test to assess how the total number of nutrients exceeding thresholds for each product relates to its price per 100 g and per 100 kcal (418 kJ). If the test indicated a statistically significant difference, we conducted a Dunn’s post hoc test to determine between which specific groups (based on the count of nutrients exceeding thresholds) the mean prices were significantly different. The Holm-Bonferroni correction was applied to adjust for multiple comparisons in all analyses, thereby reducing the risk of Type 1 error. A *P*-value of ≤ 0·05 was considered statistically significant for all analyses.

## Results

### Analytic dataset

A total of 2113 products were analysed across the different categories: 340 in sliced breads, 392 in breakfast cereals, 569 in salty snacks, 694 in cookies and 118 in processed cheeses. Details on the distribution of products within the different subcategories are shown in Table 1.

**Table 1.** Distribution of the products in the different subcategories and the number of products over or under Canadian nutrition symbol thresholds

		Saturated fat threshold		Sugars threshold		Na threshold		Total
Category	Subcategories	Below	Exceed	Below	Exceed	Below	Exceed	
Sliced breads	Overall	335	5	331	9	225	115	340
	Mixed grain	160	1	161	0	103	58	161
	Whole grain	89	0	89	0	65	24	89
	Refined grain	61	4	64	1	36	29	65
	With fruits	25	0	17	8	21	4	25
Breakfast cereals	Overall	339	53	314	78	380	12	392
	Granola	128	49	154	23	176	1	177
	Sweetened	71	0	41	30	66	5	71
	Muesli	41	3	34	10	44	0	44
	Plain	41	0	41	0	40	1	41
	Chocolate	29	1	16	14	28	2	30
	Bitesize	17	0	17	0	15	2	17
Salty snacks	Overall	492	77	560	9	338	231	569
	Chips	328	41	366	3	264	105	369
	Popped	61	8	65	4	39	10	69
	Extruded	50	11	61	0	16	45	61
	Pretzel	22	4	26	1	1	25	26
	Mixes	17	6	23	0	5	18	23
	Other	14	7	19	2	13	8	21
Cookies	Overall	238	456	264	430	692	2	694
	Regular	140	248	160	228	387	1	388
	Sandwich	23	81	9	95	104	0	104
	Wafer	14	68	21	61	82	0	82
	Tea	28	30	55	3	58	0	58
	Soft	11	26	8	29	36	1	37
	Biscotti	22	3	11	14	25	0	25
Processed cheeses	Overall	43	75	118	0	105	13	118
	Cream cheese	12	58	70	0	69	1	70
	Single-serving	27	12	39	0	31	8	39
	To spread	2	4	6	0	3	3	6
	For cooking	2	1	3	0	2	1	3

Analysis was performed on both entire food categories and their respective subcategories. However, within certain subcategories, analyses were not always possible due to the absence of products with specific nutrients exceeding their thresholds. Even when only a few products within a category exceeded the threshold for a specific nutrient, analyses were still performed, but results were interpreted with caution. Additionally, Table 1 presents the number of products in each category and subcategory that fall below or exceed the threshold for each nutrient of concern.

### Saturated fat threshold

Results for each food category as well as subcategories where significant associations were observed are presented in Table 2. In the sliced breads category, products under the saturated fat threshold had significantly lower prices per 100 g and per 100 kcal (418 kJ). This was also observed in the refined grain subcategory. A similar difference was observed in the overall breakfast cereals category, where products above the saturated fat threshold had a higher mean price per 100 g and per 100 kcal (418 kJ) compared to those under it. This difference, however, was only significant for the granola subcategory. In the salty snacks category, no significant differences were observed overall, but in the chips subcategory, products exceeding the saturated fat threshold were more expensive than those below it for both price metrics. In the processed cheeses category, no significant differences were found overall. However, single-serving processed cheeses above the saturated fat threshold were more expensive (price per 100 g and 100 kcal (418 kJ)), while cream cheeses above the saturated fat threshold had lower prices per 100 g and per 100 kcal (418 kJ). Finally, no differences in price were found in the overall cookies category, but only in two subcategories. In the tea biscuits subcategory, products over the threshold had higher prices per 100 g and per 100 kcal (418 kJ) than those under. On the contrary, sandwich cookies below the saturated fat threshold were more expensive than those above it, though this was significant only when looking at the price per 100 kcal (418 kJ).

**Table 2.** Association between the Canadian nutrition symbol saturated fat threshold and the price per 100 g and per 100 kcal (418 kJ)

			Below threshold			Exceed threshold			
Food category	Subcategories	Price metric	*n*	Mean price ($)	sd	*n*	Mean price ($)	sd	Adjusted *P*-value
Sliced breads	Overall	$/100 g	335	0·87	0·40	5	**1·42**^†^	0·43	≤ 0·01^*^
		$/100 kcal		0·35	0·18		**0·47**	0·15	0·03
	Refined grains	$/100 g	61	0·71	0·30	4	**1·38**	0·49	≤ 0·01
		$/100 kcal		0·29	0·19		**0·47**	0·18	≤ 0·01
Breakfast cereals	Overall	$/100 g	339	1·41	0·62	53	**2·10**	1·06	≤ 0·01
		$/100 kcal		0·36	0·16		**0·44**	0·22	0·02
	Granola	$/100 g	128	1·54	0·55	49	**2·13**	1·09	0·02
		$/100 kcal		0·37	0·15		0·45	0·22	0·18
Salty snacks	Overall	$/100 g	492	1·58	0·75	77	1·63	0·73	0·76
		$/100 kcal		0·33	0·18		0·31	0·14	0·92
	Chips	$/100 g	328	1·44	0·58	41	**1·81**	0·88	≤ 0·01
		$/100 kcal		0·28	0·12		**0·34**	0·16	≤ 0·01
Processed cheeses	Overall	$/100 g	43	2·12	1·66	75	2·01	0·92	0·34
		$/100 kcal		0·95	0·70		0·80	0·38	0·86
	Single-serving	$/100 g	27	1·27	0·75	12	**2·51**	1·02	≤ 0·01
		$/100 kcal		0·67	0·61		**0·99**	0·51	0·02
	Cream cheese	$/100 g	12	**3·86**	1·91	58	1·96	0·90	≤ 0·01
		$/100 kcal		**1·64**	0·47		0·78	0·36	≤ 0·01
Cookies	Overall	$/100 g	238	1·60	0·92	456	1·77	1·13	0·52
		$/100 kcal		0·37	0·21		0·36	0·23	0·52
	Sandwich	$/100 g	23	1·36	0·46	81	1·24	0·78	0·13
		$/100 kcal		**0·32**	0·11		0·25	0·15	≤ 0·01
	Tea	$/100 g	28	0·94	0·44	30	**1·73**	1·13	0·03
		$/100 kcal		0·20	0·09		**0·35**	0·22	0·03

* *P*-values are from the Wilcoxon test.

† Cells in bold are the higher mean price (*P* ≤ 0·05).

### Sugars threshold

Results are presented in Table 3. No significant association was found between price and exceeding the sugars threshold for sliced breads, both overall and within subcategories. Overall breakfast cereals, granola and sweetened cereals subcategories above the sugars threshold had a lower price per 100 g and per 100 kcal (418 kJ) compared with products below the threshold. In the salty snacks category, no significant differences were found overall, but in the chips subcategory, products below the sugars threshold had a lower price per 100 g and per 100 kcal (418 kJ), while the opposite was observed in the popped snacks subcategory. There was no statistical difference in the overall cookies category. However, sandwich and soft cookies exceeding the sugars threshold had lower prices per 100 g and per 100 kcal (418 kJ).

**Table 3.** Association between the Canadian nutrition symbol sugars threshold and the price per 100 g and per 100 kcal (418 kJ)

			Below threshold			Exceed threshold			
Food category	Subcategories	Price metric	*n*	Mean price ($)	sd	*n*	Mean price ($)	sd	Adjusted *P*-value
Sliced breads	Overall	$/100 g	331	0·88	0·41	9	0·92	0·28	0·49^*^
		$/100 kcal		0·35	0·18		0·32	0·10	0·99
Breakfast cereals	Overall	$/100 g	314	**1·61** ^†^	0·75	78	1·11	0·48	≤ 0·01
		$/100 kcal		**0·40**	0·18		0·28	0·12	≤ 0·01
	Granola	$/100 g	154	**1·79**	0·80	23	1·10	0·23	≤ 0·01
		$/100 kcal		**0·41**	0·18		0·26	0·05	≤ 0·01
	Sweetened	$/100 g	41	**1·32**	0·50	30	1·04	0·51	0·03
		$/100 kcal		**0·34**	0·13		0·26	0·13	0·03
Salty snacks	Overall	$/100 g	560	1·58	0·74	9	2·01	1·11	0·43
		$/100 kcal		0·32	0·17		0·38	0·19	0·43
	Chips	$/100 g	366	1·47	0·61	3	**2·98**	1·28	0·03
		$/100 kcal		0·29	0·12		**0·52**	0·22	0·03
	Popped	$/100 g	65	**2·09**	0·92	4	1·13	0·39	0·03
		$/100 kcal		**0·47**	0·24		0·24	0·07	0·03
Cookies	Overall	$/100 g	264	1·65	0·95	430	1·74	1·13	1·00
		$/100 kcal		0·35	0·20		0·37	0·24	1·00
	Sandwich	$/100 g	9	**1·74**	0·76	95	1·23	0·70	0·03
		$/100 kcal		**0·37**	0·15		0·26	0·14	0·03
	Soft	$/100 g	8	**2·50**	0·93	29	1·42	0·47	≤ 0·01
		$/100 kcal		**0·54**	0·21		0·37	0·18	0·02

* *P*-values are from the Wilcoxon test.

† Cells in bold are the higher mean price (*P* ≤ 0·05).

### Na threshold

Products exceeding the Na threshold in the overall sliced breads category had a significantly lower price per 100 g and per 100 kcal (418 kJ). This difference was also observed in the overall salty snacks category as well as in chips, extruded snacks and the ‘other snacks’ subcategories. No significant associations between Na and price were found in the other food categories (Table 4).

**Table 4.** Association between the Canadian nutrition symbol Na threshold and the price per 100 g and per 100 kcal (418 kJ)

			Below threshold			Exceed threshold			
Food category	Subcategories	Price metric	*n*	Mean price ($)	sd	*n*	Mean price ($)	sd	Adjusted *P*-value
Sliced breads	Overall	$/100 g	225	**0·91** ^†^	0·41	115	0·81	0·39	0·02^*^
		$/100 kcal		**0·37**	0·19		0·32	0·15	0·01
Breakfast cereals	Overall	$/100 g	380	1·52	0·73	12	1·16	0·43	0·14
		$/100 kcal		0·38	0·18		0·30	0·11	0·15
Salty snacks	Overall	$/100 g	338	**1·66**	0·77	231	1·48	0·71	0·01
		$/100 kcal		**0·34**	0·18		0·30	0·17	0·02
	Chips	$/100 g	264	**1·52**	0·63	105	1·37	0·62	0·05
		$/100 kcal		**0·30**	0·13		0·27	0·13	0·05
	Extruded	$/100 g	16	**2·36**	0·98	45	1·58	0·86	≤ 0·01
		$/100 kcal		**0·50**	0·24		0·30	0·21	≤ 0·01
	Others	$/100 g	13	**2·26**	0·86	8	1·41	0·30	0·01
		$/100 kcal		**0·51**	0·24		0·28	0·04	0·01
Processed cheeses	Overall	$/100 g	105	2·06	1·28	13	2·00	0·80	1·00
		$/100 kcal		0·85	0·52		0·88	0·56	1·00
Cookies	Overall	$/100 g	692	1·71	1·07	2	1·90	0·42	0·63
		$/100 kcal		0·37	0·22		0·43	0·02	0·63

* *P*-values are from the Wilcoxon test.

† Cells in bold are the higher mean price (*P* ≤ 0·05).

### Number of nutrients exceeding thresholds

None of the products analysed exceeded the thresholds for all three nutrients of concern. Table 5 presents the five food categories and their subcategories that showed at least one significant price difference based on the number of nutrients exceeding thresholds. Notably, results varied by food categories and even within subcategories. No significant association was found between the number of nutrients exceeding thresholds and the price of sliced breads. In the breakfast cereals category, items with two nutrients exceeding thresholds had a significantly lower price per 100 g and per 100 kcal (418 kJ) compared to items with one or no nutrient exceeding thresholds. This was also observed in the granola subcategory, although items with only one nutrient above thresholds had a significantly higher price per 100 g than those with no exceeding nutrients. In the salty snacks category, a significant association was only found in the extruded snacks subcategory, where products with one or two nutrients above thresholds had a significantly lower price per 100 g and per 100 kcal (418 kJ) than items with no exceeding nutrients. However, there was no significant price difference between products with one or two nutrients exceeding thresholds. An opposite pattern was observed in the processed cheeses category. Single-serving cheeses with one nutrient above thresholds had a significantly higher price per 100 g and per 100 kcal (418 kJ) than products with no exceeding nutrients, whereas the opposite was observed in the cream cheeses subcategory. Finally, in the cookies category, a significant association was only noted in the sandwich cookies subcategory, where products with two nutrients exceeding thresholds had a lower price per 100 kcal (418 kJ) than those with one or no nutrient exceeding thresholds.

**Table 5.** Association between the number of nutrients exceeding Canadian nutrition symbol thresholds and the mean price per 100 kcal (418 kJ) and per 100 g

		Number of nutrients exceeding thresholds						Comparisons between number of nutrients					
Food category	Subcategories	0		1		2		0 *v*. 1		0 *v*. 2		1 *v*. 2	
		Mean price ($)	sd	Mean price ($)	sd	Mean price ($)	sd	z-score	*P*-value	z-score	*P*-value	z-score	*P*-value
Sliced breads	Overall. *n* 340	*n* 215		*n* 121		*n* 4							
	$/100 kcal	0·37	0·19	0·33	0·15	0·28	0·12	1·92^*^	0·16^*^	0·82	0·82	0·39	0·70
	$/100 g	0·90	0·41	0·85	0·41	0·83	0·39	1·07	0·86	0·20	1	−0·04	0·97
Breakfast cereals	Overall. *n* 392	*n* 271		*n* 99		*n* 22							
	$/100 kcal	0·38	0·17	0·38	0·20	0·25	0·06	0·35	0·73	**4·27** ^†^	**≤ 0·01**	**3·84**	**≤ 0·01**
	$/100 g	1·48	0·62	1·67	0·98	1·05	0·27	−0·57	0·57	**3·67**	**≤ 0·01**	**3·74**	**≤ 0·01**
	Granola. *n* 177	*n* 118		*n* 45		*n* 14							
	$/100 kcal	0·38	0·15	0·47	0·22	0·25	0·05	−1·84	0·07	**3·61**	**≤ 0·01**	**4·39**	**≤ 0·01**
	$/100 g	1·57	0·55	2·21	1·09	1·12	0·25	**−2·78**	**≤ 0·01**	**3·22**	**≤ 0·01**	**4·57**	**≤ 0·01**
Salty snacks	Overall. *n* 569	*n* 291		*n* 239		*n* 39							
	$/100 kcal	0·33	0·18	0·32	0·17	0·28	0·10	1·42	0·47	1·01	0·62	0·28	0·78
	$/100 g	1·64	0·75	1·54	0·77	1·51	0·61	1·96	0·15	0·66	1	−0·33	0·74
	Extruded. *n* 61	*n* 14		*n* 38		*n* 9							
	$/100 kcal	0·51	0·24	0·34	0·22	0·21	0·05	**2·49**	**0·03**	**3·04**	**≤ 0·01**	1·41	0·16
	$/100 g	2·41	1·00	1·70	0·91	1·14	0·27	**2·43**	**0·03**	**3·10**	**≤ 0·01**	1·52	0·13
Processed cheeses	Overall. *n* 118	*n* 41		*n* 66		*n* 11							
	$/100 kcal	0·90	0·67	0·86	0·45	0·67	0·25	−0·96	1	0·19	0·85	0·78	0·87
	$/100 g	2·08	1·69	2·08	0·95	1·81	0·73	−1·90	0·17	−0·61	1	0·53	0·60
	Single-serving. *n* 39	*n* 25		*n* 8		*n* 6							
	$/100 kcal	0·56	0·48	1·49	0·53	0·66	0·30	**−3·68**	**≤ 0·01**	−1·06	0·29	1·87	0·12
	$/100 g	1·14	0·58	3·07	0·66	1·91	0·92	**−4·15**	**≤ 0·01**	−1·51	0·13	1·85	0·13
	Cream cheeses. *n* 70	*n* 12		*n* 57		*n* 1							
	$/100 kcal	1·64	0·47	0·77	0·36	1·04		**4·96**	**≤ 0·01**	0·82	0·83	−0·72	0·47
	$/100 g	3·86	1·91	1·95	0·91	2·42		**4·17**	**≤ 0·01**	0·62	0·54	−0·67	1
Cookies	Overall. *n* 694	*n* 125		*n* 250		*n* 319							
	$/100 kcal	0·33	0·17	0·39	0·23	0·36	0·23	−1·28	0·1	−0·08	0·94	1·56	0·35
	$/100 g	1·49	0·74	1·77	1·08	1·75	1·15	−1·47	0·43	−1·05	0·59	0·59	0·55
	Sandwich. *n* 104	*n* 3		*n* 26		*n* 75							
	$/100 kcal	0·44	0·16	0·31	0·11	0·25	0·14	1·19	0·23	**2·40**	**0·03**	**3·01**	**≤ 0·01**
	$/100 g	1·99	0·87	1·35	0·46	1·21	0·77	1·52	0·13	2·2	0·08	1·63	0·21

* z-scores and *P*-values are from the Dunn’s post hoc test.

† Cells in bold indicate statistically significant differences (*P* ≤ 0·05).

## Discussion

This study compared the prices of food products according to their nutritional quality, using the FOPL criteria from the upcoming Canadian nutrition policy that will be mandatory by 2026. No consistent association between the price of the products and their nutritional quality was observed. Instead, this association appeared to be mediated by the type of nutrients analysed, with variations observed across the different food categories.

Within a given category, items that would have a ‘high in saturated fat’ nutrition symbol were generally more expensive. At first glance, this finding seems surprising as it was previously demonstrated that higher saturated fat consumption is associated with lower diet costs^(19)^. This discrepancy likely arises because prior studies analysed overall diet costs rather than comparing the prices of products within specific food categories. For instance, fruits and vegetables, typically low in saturated fat, contribute considerably to the overall diet cost, thereby influencing previous findings^(19,30,31)^. In the present study, it is suggested that, within the same food categories, the higher price of some products with greater saturated fat content is likely because certain ingredients high in saturated fat tend to be more expensive. For instance, in the granola subcategory (which constituted a significant part of our breakfast cereals sample: 177/392, 45·2 %), products exceeding the saturated fat threshold were more expensive compared to those below this threshold. These cereals typically are made with oil, butter, nuts and/or seeds, ingredients with a higher fat content and that are more expensive than those commonly found in breakfast cereals, such as grains, sweeteners and salt^(24,32)^. In contrast, we observed the opposite trend for cream cheese products. One possible explanation for this observation could be that ‘low-fat’ or ‘light’ versions of products are sometimes perceived as more premium by consumers than their ‘regular’ counterparts, making them more willing to pay higher prices, which could result in higher market prices in the same food category^(33)^. In summary, within a food category, products higher in saturated fat are often more expensive as they are made with ingredients rich in saturated fat that are also higher in price.

Conversely, products within the same food category categorised as ‘high in sugars’ according to the sugars threshold of the upcoming nutrition symbol were generally less expensive than those not meeting this criterion. This finding was mainly seen in breakfast cereals and cookies, which were identified as having many products with a higher sugars content^(5)^. Similar findings were also reported in Portugal, where sugars content in breakfast cereals was negatively correlated with price^(34)^. One possible explanation is that sugars has historically been an inexpensive ingredient with multifunctional properties beyond sweetness, including enhancing texture, aiding preservation and improving flavor^(35)^. Consequently, producing lower-sugar alternatives may require more expensive ingredients and more complex processes to achieve acceptable products for the consumer^(35)^. This disparity in food pricing linked to sugars content is of concern, as it may complicate healthier food choices, given that price is known to influence consumer food choices^(36)^. However, discrepancies were observed in salty snacks, where chips that exceeded the sugars threshold were more expensive than those below this threshold. Salty snacks generally do not contain significant amounts of sugars^(21)^. In our sample, only 9 out of the 569 products (1·6 %) had a sugars content higher than 15 % DV and would then require a sugars nutrition symbol. Therefore, the sample is too small to draw definitive conclusions. However, it can still be hypothesised that the opposite trend observed in chips could be explained by their ingredients. Indeed, the three chip products that were above the sugars threshold were either made from beets, coconut, or banana, all of which have higher sugars content than regular potato chips and, as we previously explained, are more expensive^(22)^.

Overall salty snacks and sliced breads that exceed the Na threshold were significantly less expensive than products with lower Na content. This association was also evident across three salty snack subcategories: chips, extruded snacks and others. For many consumers, salty taste is an important factor driving the acceptability of salty snacks, necessitating Na replacement to maintain consumer preference^(37)^. Decreasing Na content in food products impacts their cost, as salt is a very inexpensive ingredient, while its substitutes are often costlier^(38)^. It was estimated that reducing Na by 20 to 30 % could increase costs by 5 to 30 %, depending on the product^(38)^. Also, our findings are consistent with our previous observations where salty snacks with a more natural profile had lower Na content but were priced higher than regular potato chips^(22)^. Other food categories analysed had few products with Na content exceeding 15 % of the DV, possibly explaining the absence of observed price associations.

Statistically significant associations were observed between the number of nutrients of concern exceeding thresholds and product prices across the different food categories. Generally, products with two nutrients exceeding thresholds tended to cost significantly less than products with only one or no nutrients exceeding thresholds. This aligns with prior research describing less nutritious products, such as those with high amounts of added sugars and saturated fat, as being cheaper^(19,39)^. However, variations were observed among food categories; for example, single-serving processed cheeses and granola products with only one nutrient exceeding thresholds cost more than products with no nutrient above thresholds, while the opposite trend was noted for cream cheeses and extruded salty snacks. The differences between food categories had previously been described by Katz et al. who also reported discrepancies among food categories regarding nutritional value and pricing^(40)^. In this analysis, such discrepancies are likely due to saturated fat, which was often associated with higher prices in these subcategories. These results highlight the complexity of the association between food pricing and nutritional content, demonstrating the necessity to study food categories individually to fully understand the factors affecting food pricing.

Most significant associations were observed for both price per 100 g and price per 100 kcal (418 kJ) metrics. However, it was previously demonstrated that the association between nutritional quality and food price is influenced by the price metric used. For instance, vegetables, rich in nutrients but low in calories, might appear expensive when evaluated by price per 100 kcal (418 kJ) but not when looking at price per 100 g^(41)^. This distinction is crucial when assessing the overall cost of the diet as it may differ due to the varying product compositions. However, it is believed that when comparing food within the same food category, this difference becomes less important as products tend to have a more uniform composition. Therefore, it is likely that by looking at price per 100 g and per 100 kcal (418 kJ) within specific food categories, the variation seen in broader dietary comparisons was minimised, leading to similar findings across both metrics. Moreover, it is important to note that the thresholds from the Canadian nutrition symbol were used in this study to assess nutritional quality. Since two of the three nutrients featured on the nutrition symbol are high in calories (sugars and saturated fat), the presence of the nutrition symbol is likely correlated with calorie content^(42)^.

These findings have significant implications for public health. It was observed that the association between Na, saturated fat and sugars content and food prices is not straightforward, as it is not solely tied to the quantity of these nutrients but also to the ingredients and the processing methods used. This aligns with Aceves-Martins et al.’s findings, which noted that the nutritional quality and cost of UPFs are lower compared to minimally processed fresh foods, regardless of their sugars, saturated fat and Na content^(43)^. This highlights the challenge of Canada’s upcoming mandatory nutrition symbol implementation concerning food inequities. Indeed, potential reformulation to avoid warning labels could lead to price increases^(12)^. This may encourage the purchase of less healthy options, particularly for price-sensitive consumers, who are often of lower socioeconomic status, exacerbating health inequalities^(44)^. In addition, it is important to note that consumers, particularly children, are inclined to prefer salty, sweet and high-fat foods due to their high palatability^(19,37)^. When combined with lower prices, these preferences can become a powerful driver of food choices^(19)^. An umbrella review on the impact of food environment policies on socioeconomic inequalities also revealed inconclusive results regarding FOPL, as the few identified reviews showed either no effect or inconsistent outcomes on this issue^(45)^. Therefore, to effectively reduce health inequities, FOPL should be considered in conjunction with other food environmental policies that can work synergistically to address these inequities^(46)^. For example, although results remain mixed, subsidies for more nutritious foods and taxes on less nutritious ones have shown positive effects on dietary intake in some contexts, particularly among lower socioeconomic groups^(45)^. Additionally, while combining FOPL with mandatory reformulation policies could be effective, it is crucial to develop policies encouraging producers to improve their products’ nutritional quality without raising consumer prices, ensuring that these improvements also benefit lower socioeconomic groups^(45)^. For these policies to have a meaningful effect on public health, they must be systematically implemented across most food categories, including those most consumed by lower socioeconomic groups^(45)^. To further support this argument, the WHO emphasises the importance for Member States to address the commercial determinants of health (CDoH), for example, through fiscal policies and government regulations on advertising, promotion and sponsorship of harmful products, to improve both health and equity^(47)^.

This study has several strengths. First, it provides a comprehensive snapshot of the breakfast cereals, sliced breads, processed cheeses, cookies and salty snacks in Québec (Canada) by including all items available at the time of sampling. Second, to our knowledge, the nutrition symbol was broken down into its components for the first time to analyse specific nutrients’ association with price. Third, food categories were also divided into subcategories of similar products to enable more detailed analysis, as products within the same category often vary widely in composition. Lastly, all analyses were conducted using two different price metrics to assess potential variations in results between them.

Nonetheless, the study has some limitations. The offerings in a food category can rapidly change with the introduction of new items, compositional changes, or price fluctuations. However, our data reflects the market at a specific time point and does not account for price variations between the different stores in Québec City or promotional prices, nor are our price data weighted according to demand for each location. Nevertheless, our price data were validated for two of the five categories by merging it with NielsenIQ sales data of products sold in Québec over 52 weeks for breakfast cereals (June 2020–June 2021) and sliced breads (January 2021–January 2022) using unique product codes (UPC). Validation was limited to these two food categories as NielsenIQ sales data were only available for them due to purchase constraints. We observed no statistically significant price differences between our data and NielsenIQ data. Moreover, our dataset does not include products without a UPC, which are typically less processed and may have better nutritional composition but higher prices^(48)^. For instance, this exclusion includes in-store prepared products, which can be found across all five food categories analysed. Although we cannot determine their exact number, we were able to establish that the dataset for each food category covers approximately 79 % of sales volume and 77 % of products in those categories in Quebec and Canada, based on the NielsenIQ database^(5)^. Additionally, the analyses of this study are based on thresholds of the Canadian FOP nutrition symbol, which is not yet mandatory. Consequently, it cannot be predicted whether the results would differ post-implementation due to potential industry reformulations to avoid labeling, which could impact product prices. Future studies should reassess the association between nutrition symbols and prices after the legislation is enacted to document its impact on the food environment. Moreover, our study used the Canadian FOP nutrition symbol to assess nutritional quality^(10)^. While this tool is appropriate given its upcoming mandatory implementation in Canada it has limitations as it solely evaluates three nutrients of concern and not the overall nutritional quality, which should also consider beneficial nutrients such as fiber and protein^(49)^. Indeed, nutrition warning symbol is useful for identifying products high in certain nutrients of concern, while other FOP labels have been shown to better assess the overall nutritional quality of products^(49)^. For instance, a product containing nuts could be identified as high in saturated fat, despite nuts being nutrient-dense and not necessarily indicating poor nutritional quality^(50)^. Including a broader range of nutrients, particularly a balance of both nutrients to limit and those to encourage, could offer a more comprehensive assessment of nutritional quality. It would also have been interesting to analyse whether the patterns observed for nutrients to limit would also be seen for beneficial nutrients. For example, fiber-rich sliced breads are likely more expensive due to higher grain content^(26,32)^. Lastly, the present study focused on only five food categories that were selected from the original fifteen food categories by the Food Quality Observatory as they were identified as the ones contributing the most to Na, sugars, or saturated fat purchases among the Québec population^(5)^. That said, given that our results showed the association between nutritional quality and price varies across food categories, it would have been relevant to include other major contributors to these three nutrients of concern, such as sugary drinks for sugars to provide a more comprehensive picture. However, these categories were not part of the original sample.

In conclusion, this study shows that products containing nutrients of concern that would currently exceed thresholds according to Health Canada’s criteria are overall associated with lower prices within the same food category, except for foods that would be labeled as ‘high in’ saturated fat. Nonetheless, this association is complex and highly mediated by the types of nutrients and products analysed. These findings highlight the intricate interaction between food nutritional content, composition and pricing. As Canada prepares to implement its nutrition symbol^(10)^, monitoring its impact on food prices is crucial, particularly to prevent aggravating health inequalities. Future research should reassess the association between the nutrition symbol and food prices post-implementation. Meanwhile, efforts are needed to help producers improve the nutritional quality of their products without raising food prices.

## Supporting information

Petitclerc et al. supplementary material Petitclerc et al. supplementary material

## Supplementary material

For supplementary material accompanying this paper visit https://doi.org/10.1017/S1368980025100797
