# Supplementary material for: Association between the nutritional quality of Canadian packaged foods and their prices: an analysis across five food categories
Source: Public Health Nutr. 2025 Aug 13;28(1):e135. doi: 10.1017/S1368980025100797 (PMC12465062; doi:10.1017/S1368980025100797)
Supplement: Petitclerc et al. supplementary material [file S1368980025100797sup001.docx]

**Supplementary material**

Definitions of food category subcategories

| **Category** | **Subcategories** | **Definition** |
| --- | --- | --- |
| **Sliced breads** | **Mixed grain** | Bread made of a single type of grain, partly whole grain and partly refined grain (e.g., whole wheat and fortified flour bread). |
|  | **Whole grain** | Bread made from 100% whole grain (e.g., whole wheat flour bread). |
|  | **Refined grain** | Bread made from 100% refined grain (e.g., enriched wheat flour bread (white)). |
|  | **With fruits** | Fruit or vegetable bread. |
| **Breakfast cereals** | **Granola** | Muesli containing crispy nuggets (the grains are cooked) and ingredients other than grain (e.g. nuts, dried fruit, chocolate). |
|  | **Sweetened** | Caramel, honey or other sweet flavored. |
|  | **Muesli** | Muesli without crispy nuggets (the grains are not cooked) and containing ingredients other than cereal grains (e.g., nuts, dried fruit, chocolate). |
|  | **Plain** | Corn flakes and other unsweetened cereals. May have flavors (e.g., cinnamon, vanilla) except chocolate. |
|  | **Chocolate** | Chocolate flavored. |
|  | **Bitesize** | Unsweetened wheat, corn or whole rice shaped in bites. |
| **Salty snacks** | **Chips** | Chips-style snack. No added air. Includes coconut, banana, and apple "chips. |
|  | **Popped** | Puffed or popped-style snack. Includes puffed rice and popcorn. |
|  | **Extruded** | Extruded-style snack, typically in the shape of sticks or rings. May be labeled as "puffed" on the packaging. |
|  | **Pretzel** | Pretzel-style snack. |
|  | **Mixes** | Mix of the above-mentioned products. |
|  | **Other** | Other snacks (e.g., whole legumes, sesame sticks). Excludes coconut, banana, and apple chips. |
| **Cookies** | **Regular** | Regular cookie with or without coating/filling. Excludes tea cookies. (e.g., ladyfingers, Graham crackers, and marshmallow cookies). |
|  | **Sandwich** | Two cookies sandwiched with filling inside. |
|  | **Wafer** | Small flaky dry waffle, cookie sticks (pirouline, straws), and "crêpêttes". |
|  | **Tea** | Butter cookie. Includes tea cookies, shortbread, or digestive cookies. Must be plain (without candy, fruits, or nuts) and without coating. Includes cookies with nut extracts and flavors. |
|  | **Soft** | Cookie in the form of a cake, brownie, or patty. Excludes cake- or brownie-flavored cookies. Includes coconut soft cookies. |
|  | **Biscotti** | Twice-baked cookie, such as biscotti. |
| **Processed cheeses** | **Cream cheese** | Cream cheese or cream cheese product. Includes plant-based alternatives. |
|  | **Single-serving** | Processed cheese product, typically presented in slices or triangles for individual consumption. Includes plant-based alternatives. |
|  | **To spread** | Spreadable processed cheese product. Includes cheese spreads and plant-based alternatives. |
|  | **For cooking** | Processed cheese product, typically presented in a block and often used for cooking. Includes plant-based alternatives. Excludes products labeled "cream cheese. |
